# Inhibition of collagen XI alpha 1-induced fatty acid oxidation triggers apoptotic cell death in cisplatin-resistant ovarian cancer

**DOI:** 10.1038/s41419-020-2442-z

**Published:** 2020-04-20

**Authors:** Sameera Nallanthighal, Miran Rada, James Patrick Heiserman, Jennifer Cha, Jessica Sage, Bo Zhou, Wei Yang, Ye Hu, Chaitali Korgaonkar, Christina Terpsithea Hanos, Zahra Ashkavand, Kenneth Norman, Sandra Orsulic, Dong-Joo Cheon

**Affiliations:** 1 Department of Regenerative and Cancer Cell Biology, Albany Medical College, Albany, NY 12208 USA; 2 Cancer Biology Program, Samuel Oschin Comprehensive Cancer Institute, Cedars-Sinai Medical Center, Los Angeles, CA 90048 USA; 3 Women’s Cancer Program, Samuel Oschin Comprehensive Cancer Institute, Cedar-Sinai Medical Center, Los Angeles, CA 90048 USA; 4 Department of Obstetrics and Gynecology, Albany Medical College, Albany, NY 12208 USA; 5 Department of Obstetrics and Gynecology, David Geffen School of Medicine, University of California, Los Angeles, CA 90095 USA

**Keywords:** Cancer, Cancer

## Abstract

Collagen type XI alpha 1 (COL11A1) is a novel biomarker associated with cisplatin resistance in ovarian cancer. However, the mechanisms underlying how COL11A1 confers cisplatin resistance in ovarian cancer are poorly understood. We identified that fatty acid β-oxidation (FAO) is upregulated by COL11A1 in ovarian cancer cells and that COL11A1-driven cisplatin resistance can be abrogated by inhibition of FAO. Furthermore, our results demonstrate that COL11A1 also enhances the expression of proteins involved in fatty acid synthesis. Interestingly, COL11A1-induced upregulation of fatty acid synthesis and FAO is modulated by the same signaling molecules. We identified that binding of COL11A1 to its receptors, α1β1 integrin and discoidin domain receptor 2 (DDR2), activates Src-Akt-AMPK signaling to increase the expression of both fatty acid synthesis and oxidation enzymes, although DDR2 seems to be the predominant receptor. Inhibition of fatty acid synthesis downregulates FAO despite the presence of COL11A1, suggesting that fatty acid synthesis might be a driver of FAO in ovarian cancer cells. Taken together, our results suggest that COL11A1 upregulates fatty acid metabolism in ovarian cancer cells in a DDR2-Src-Akt-AMPK dependent manner. Therefore, we propose that blocking FAO might serve as a promising therapeutic target to treat ovarian cancer, particularly cisplatin-resistant recurrent ovarian cancers which typically express high levels of COL11A1.

## Introduction

The Warburg effect describes a phenomenon where cancer cells rely on glycolysis for their energy needs even in the presence of oxygen^1^. However, increasing evidence suggests that other metabolic pathways also play important roles in supplying energy and biomass to cancer cells^2–4^. For example, rapidly proliferating cancer cells rely on large amounts of fatty acids to support various biological processes including membrane formation and signaling. Thus, aberrant fatty acid metabolism has been implicated in driving malignancy of several cancers, such as breast, prostate, leukemia, and ovarian cancer^5–9^. Overexpression of fatty acid synthase (FASN), a key fatty acid synthesis enzyme, has also been reported in several cancer types and associated with poor prognosis and resistance to chemotherapy^8–16^. In addition to synthesis, mitochondrial fatty acid oxidation (FAO) seems to be important for maintaining cancer cell survival. FAO breaks down fatty acids to produce excess ATP and NADPH to support cell survival. FAO is initiated by the conversion of the long-chain fatty acids into fatty acyl-CoAs by the action of ACSL1 enzyme followed by transport into the inner mitochondrial membrane through the activity of carnitine palmitoyl transferases, CPT1 and CPT2. In the mitochondria, FAO is characterized by a series of breakdown reactions catalyzed by four major enzymes encoded by ACADM, ECHS1, HADH/HADHA/HADHB, and ACAA2, which results in the generation of acetyl-CoA, NADH, and FADH_2_. Recent studies have shown that blocking of FAO inhibits tumor cell proliferation and induces apoptosis in leukemia, myeloma, glioma, glioblastoma, prostate, breast, and ovarian cancer^6,17–22^. Overall, fatty acid metabolism is particularly important for ovarian cancer cells as they frequently disseminate to fat-rich omentum and uptake fatty acids for their growth and survival^23,24^. However, the molecular mechanisms by which ovarian cancer cells switch their metabolic phenotype to promote fatty acid metabolism and chemotherapy resistance are largely unknown.

Collagens are the major structural component of the tumor microenvironment and have emerged as an important contributor to cancer cell chemoresistance. Collagen type XI alpha 1 (COL11A1), a minor fibrillar collagen crucial for skeletal development and collagen fiber assembly, is a novel biomarker associated with poor survival and chemoresistance in several cancer types including ovarian cancer^25–29^. COL11A1 expression is increased during ovarian cancer progression with the highest expression in cisplatin-resistant recurrent tumors^27^. COL11A1 is expressed and secreted by a subset of cancer-associated fibroblasts (CAFs) adjacent to tumor cells and a small number of cancer cells including A2780cis cisplatin-resistant ovarian cancer cell line^25–27,30^. We have previously shown that COL11A1 confers cisplatin resistance by engaging α1β1 integrin and Discoidin domain receptor 2 (DDR2) on ovarian cancer cells to activate c-Src-Akt-NFkB signaling to induce inhibitor of apoptosis proteins (IAPs)^31^. Here, we report another mechanism by which COL11A1 confers cisplatin resistance by regulating ovarian cancer cell metabolism. We show that COL11A1 upregulates both fatty acid synthesis and oxidation predominantly through DDR2-Src-Akt-AMPK dependent signaling to inhibit cisplatin-induced apoptosis in ovarian cancer cells. Our results provide novel therapeutic strategies to treat cisplatin-resistant recurrent ovarian cancers which typically express high levels of COL11A1.

## Materials and methods

### Cell lines

ES2 and OVCAR3 ovarian cancer cell lines and A204 cell line were purchased from ATCC. A2780 and A2780cis ovarian cancer cell lines were purchased from SIGMA. Lenti-X 293T cells were purchased from Clontech. Human CAFs were a generous gift from Dr. Nikki Cheng (The University of Kansas, Lawrence, USA). A204, CAFs, ES2, and Lenti-X 293T cell lines were cultured in DMEM (Gibco Life Technologies) supplemented with 10% FBS (Sigma-Aldrich) and 1× penicillin/streptomycin (Gibco Life Technologies). OVCAR3, A2780, and A2780cis cell lines were cultured in RPMI (Gibco Life Technologies) supplemented with 10% FBS, 1× penicillin/streptomycin. All cells were cultured at 37 °C in 5% CO_2_. All cell lines were authenticated and confirmed to be mycoplasma free.

### Cell culture

Cancer cells were serum starved overnight prior to the experiments. Tissue culture plates were coated with 5 µg/ml of type I collagen (COL1; Gibco Life Technologies) or COL11A1 extracted from A204-scrambled cells or A204 shCOL11A1 cells^31^ (Fig. S1B) for 2 h at 37 °C. The A204 cell line is a rhabdomyosarcoma cell line that has been previously shown to express and secrete high levels of COL11A1 and very low levels of COL5A2^32^. Cancer cells were trypsinized, resuspended in 1% FBS containing medium and cultured in collagen-coated plates for 72 h before the analysis. For co-culture, CAFs were seeded in 6-well inserts (Falcon #353090) and ovarian cancer cells were plated in the companion 6-well plates (Falcon #353502) in media supplemented with 1% FBS for 72 h. CAFs have been shown to express high levels of COL11A1 and co-culture with these CAFs increased cisplatin resistance in ovarian cancer cell lines^25,27,31^. For drug treatments, cancer cells were serum starved and plated on COL11A1 extract for 48 h in media containing 1% FBS and treated with drugs (dasatinib, LY294002, MK2206 and dorsomorphin) for another 48 h. For cisplatin treatment, cancer cells were serum starved and cultured on uncoated plates, COL11A1-positive extract or COL11A1-negative extract in media containing 1% FBS for 24 h and treated with cisplatin for another 72 h.

### Cell viability assays

Cell viability was measured by CellTiter-Glo luminescent cell viability assay (Promega # G7572) using a Glomax Explorer Microplate Reader (Promega), according to the manufacturer’s protocols. For the propidium iodide (PI) staining, cancer cells were collected, washed with phosphate-buffered saline (PBS) and resuspended in PBS containing 1% FBS, 50 µg/ml PI (Sigma-Aldrich #P4170) and 10 µg/ml RNase A (Omega bio-tek #AC118). Flow cytometry analysis was performed using Becton Dickinson DXP flow cytometer and the data were analyzed by FlowJo software version 7.5 (Tree Star Inc., Ashland, OR, USA).

### Lentiviral shRNA knockdown

Stable gene knockdown was performed using short hairpin RNAs (shRNAs) as described previously^31^. pCMV-ΔR 8.2 (Addgene #8455), pCMV-VSVG (Addgene #8454), and a lentiviral construct containing shRNA (Supplementary Table 1) were co-transfected into Lenti-X 293T cells using Lipofectamine 2000 (Life technologies) according to manufacturer’s protocol. Lentivirus-containing media was harvested and filtered through a 0.45 µm polyvinylidene fluoride (PVDF) low protein-binding membrane filter (Celltreat) and mixed with polybrene (8 µg/ml). Cancer cells were incubated in the lentivirus-containing medium for 72 h at 37 °C in 5% CO_2_ and then replaced with complete growth media. Transduced cells were selected by puromycin (5 µg/ml) treatment for 72 h and gene knockdown in these cells was confirmed by Western blotting.

### Quantitative proteomic analysis

The tandem mass tag (TMT) labeling and liquid chromatography–tandem mass spectrometry (LC–MS/MS) analysis were performed by the Cedars-Sinai Mass Spectrometry and Biomarker Discovery core as described^33^. Briefly, proteins were extracted from cell pellets using lysis buffer containing 80 mM Tris–HCl, 4% sodium dodecyl sulfate, 100 mM DTT, pH 7.4, alkylated with iodoacetamide and digested with trypsin using the filter-aided sample preparation method^34^. Samples were then labeled with the TMT6plex Reagents (Thermo Scientific), mixed, and fractionated using the poly styrenedivinylbenzene reverse phase sulfonate approach^35^. Peptide fractions were separated using a 50 cm EASY-Spray analytical column on an EASY nLC 1000 ultraperformance LC system and analyzed by an LTQ Orbitrap Elite mass spectrometer (Thermo Scientific). Mass spectra were acquired in a data-dependent manner, selecting up to 15 most abundant precursor ions for higher-energy collision dissociation. Database searching was performed using Proteome Discoverer (v1.4) using the SEQUEST algorithm. Searching parameters were as follows: trypsin (Full); up to two missed cleavage; precursor ion tolerance of 10 ppm; fragment ion tolerance of 0.02 Da; carbamidomethylation of cysteines and TMT6plex modification of lysines and peptide N-term as fixed modifications; acetylation of protein N-term, oxidation of methionines and deamidation of asparagines and glutamines as variable modifications. A stringent 1% false-discovery rate (FDR) was set to filter peptide and protein identifications. The mass spectrometry proteomics data have been deposited to the ProteomeXchange Consortium via the PRIDE^36^ partner repository with the dataset identifier PXD016244. Differentially expressed proteins (DEPs) were identified based on the criterion of log2-transformed fold changes >0.5 in both replicates. Ingenuity Pathway Analysis (IPA) was applied to identify significantly changed biological pathways.

### qRT-PCR and Immunoblotting

Total RNA was extracted from cells using the RNeasy mini kit (Qiagen) and 1 µg of total RNA was reverse transcribed into cDNA using the Quantitect Reverse Transcription Kit (Qiagen). Fifty nanograms of cDNA was combined with primers and iQSYBR-Green Supermix (BioRad). The quantitative real-time polymerase chain reaction (qRT-PCR) reaction was performed using a CFX96 RT-PCR detection system (BioRad) and the data were analyzed by the 2^−ΔΔC(T)^ method. Primer sequences used for these experiments are listed in Supplementary Table 3.

For Western blotting, total protein was extracted from cells using RIPA cell lysis buffer (SIGMA) containing protease and phosphatase inhibitors (Roche). Total protein was quantified by a BCA assay kit (Pierce). Equal amounts of protein were loaded for each sample and resolved on a 4–20% Mini-PROTEAN^®^ TGX^™^ Protein Gel (BioRad). Proteins were transferred onto PVDF membranes and probed with antibodies listed in Supplementary Table 2.

### Seahorse XF FAO and glycolysis assays

The Seahorse XF FAO assay and the XF glycolysis stress assay were performed using a Seahorse XFp analyzer (Agilent) according to the manufacturer’s protocols. Briefly, 50,000 ovarian cancer cells were cultured in COL11A1-coated (for ES2) or uncoated (for A2780cis) XFp cell culture plates in serum-free media overnight. For the FAO assay, the cells were supplemented with palmitate followed by sequential injections of oligomycin (1 µM) at 20 min, phenylhydrazone (FCCP; 0.125 µM) at 30 min and rotenone/antimycin (0.5 µM) at 60 min after palmitate addition and the real-time oxygen consumption rate (OCR) was measured at different time points. To measure FAO after CD36 blockade, cells were incubated with IgA isotype control or 2 µg/ml CD36 blocking antibody^37^ (# AB23680, Abcam) overnight followed by washing to remove excess antibody. Next, oligomycin (1 µM), FCCP (0.125 µM), and rotenone/antimycin (0.5 µM) were sequentially injected and OCR is measured over time. For the glycolysis stress assay, the cells were incubated in medium without glucose or pyruvate, followed by sequential injections of glucose (10 mM), oligomycin (1 µM), and 2-deoxy glucose (2-DG; 50 mM) and the extracellular acidification rate (ECAR) was measured at different time points.

### Measurement of cellular NADH and ATP

The NAD/NADH-Glo^™^ Assay and Cell Titer-Glo assay (Promega) were performed to measure the levels of cellular NADH and ATP respectively, according to the manufacturer’s instructions. Briefly, 5000 ES2 cells were cultured in COL11A1-coated plates or PBS-coated plates for 48 h and NAD/NADH-Glo^™^ or CellTiter-Glo detection reagent was added to each well in 1:1 ratio. Luminescence was recorded over time using a Glomax Explorer Microplate Reader (Promega) and was normalized to total protein content.

### Patient data analysis

Kaplan–Meier plots were generated by the KM plotter (www.kmplot.com) using the overall survival data of 1207 ovarian cancer patients expressing high or low mean expression of mitochondrial FAO genes (CPT1A, HADHA, and ACAA2). The protein expression of CPT1A in normal ovaries and ovarian cancer was extracted from the Human Protein Atlas. Tissue microarray (TMA) and corresponding clinical data were obtained from Dr. Sandra Orsulic under an approved IRB protocol (Cedars-Sinai Medical Center). The clinical characteristics of 21 patient tumors used in TMA sections are shown in Supplementary table 5. COL11A1 in situ hybridization was performed as described^25^. We have previously shown high concordance between COL11A1 staining by in situ hybridization and immunohistochemistry and determined that in situ hybridization is more reliable in precut slides^27^. Immunostaining with a CPT1A antibody was performed by the histology core (Albany Medical College) and visualized using DAB (3,3′-Diaminobenzidine) as a chromogen. Nanozoomer (Hamamatsu) was used to acquire complete images of stained TMA and analyzed in a blinded fashion by at least three independent investigators. Kaplan–Meier curves of ovarian cancer patients with increased CPT1A expression during recurrence were plotted using GraphPad Prism software version 7.0 (GraphPad Software, La Jolla, CA, USA).

### Statistical analysis

Statistical analyses were performed using GraphPad Prism software version 7.0 (GraphPad Software, La Jolla, CA, USA). One-way ANOVA was applied to compare the mean difference of two or more groups. Mantel Cox (log rank) test was used to compare significance between survival curves. *P* ≤ 0.05 was considered significant.

## Results

### COL11A1 induces FAO in ovarian cancer cells

**Fig. 1:**
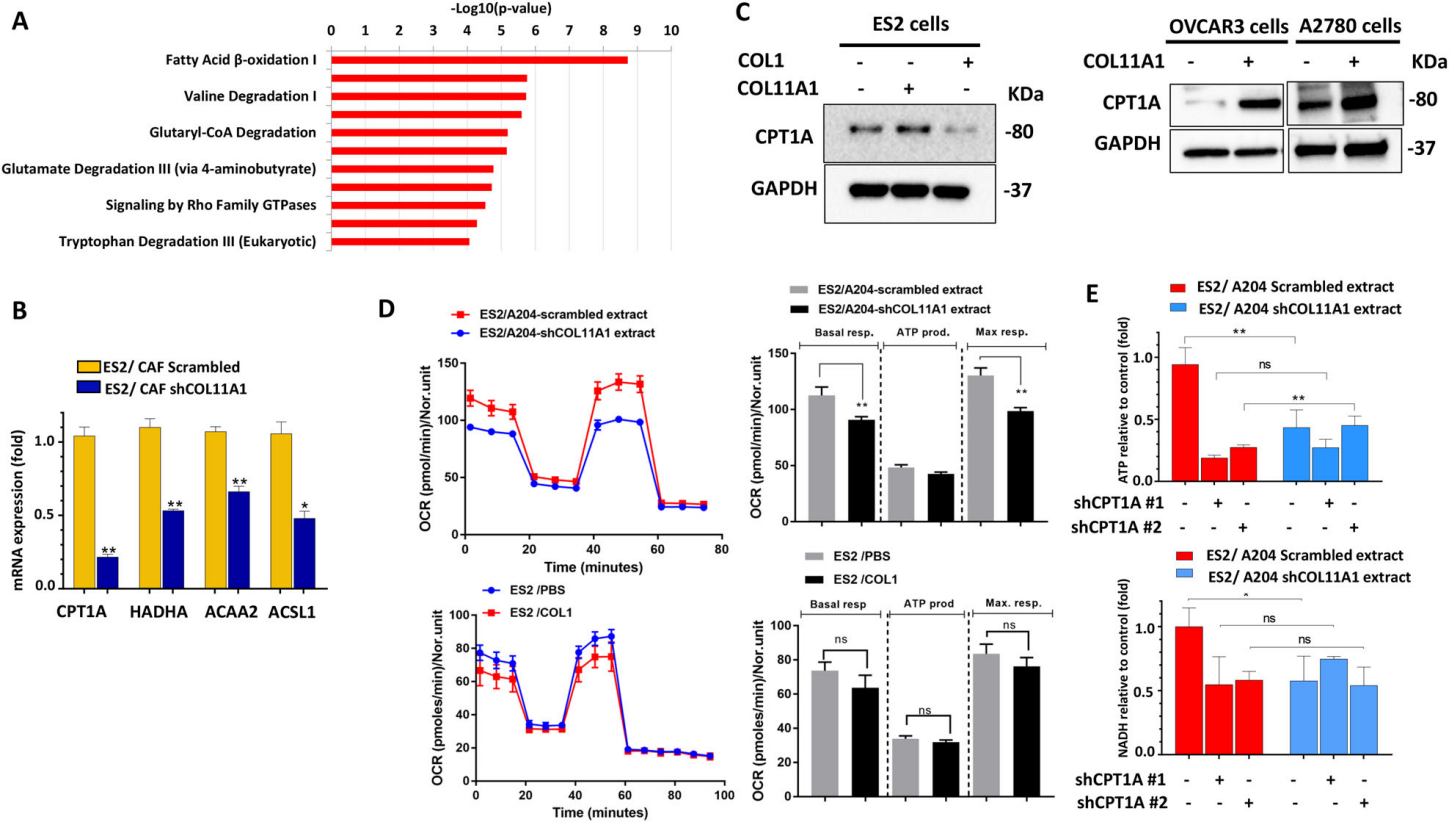
COL11A1 upregulates FAO in ovarian cancer cells. **a** Mass spectrometry analysis showing top significantly upregulated pathways in COL11A1-overexpressing, cisplatin-resistant ovarian cancer (A2780cis) cells compared to COL11A1-low, cisplatin-sensitive ovarian cancer cells (A2780). **b** Real-time PCR of FAO genes in ES2 cells co-cultured with CAFs expressing scrambled vector or shCOL11A1. The mRNA expression was normalized to RPL32. *N* = 3; *y*-axis, relative mRNA expression (fold change); error bars, SD; *p < 0.05; **p < 0.01. **c** Western blot of CPT1A in ES2 cells cultured in PBS, type I collagen (COL1)- or COL11A1-coated plates (left) and OVCAR3 and A2780 cells cultured in PBS or COL11A1-coated plates (right). GAPDH was used as a loading control. *N* = 3. **d** A real-time oxygen consumption rate (OCR) in response to sequential treatments with palmitate, oligomycin, FCCP, and antimycin-A/rotenone in ES2 cells cultured in XFp plates coated with COL11A1-positive or COL11A1-negative matrix (top) or ES2 cells cultured in PBS or COL1-coated XFp plates (bottom). Quantification of the OCR is showed on the right. *N* = 3; *y*-axis, OCR; error bar, SD; ns not significant; **p < 0.01. **e** Relative ATP (top) and NADH (bottom) levels in ES2 cells expressing scrambled or shCPT1A cultured in either COL11A1-positive or COL11A1 negative extract. *N* = 3; *y*-axis, fold change; error bars, SD; ns not significant; *p < 0.05; **p < 0.01.

To obtain mechanistic insight underlying COL11A1-mediated cisplatin resistance, we performed TMT-based quantitative proteomic analysis to compare the protein expression profiles of A2780 ovarian cancer cell line and A2780cis, a cisplatin-resistant variant of A2780 cell line which expresses high levels of COL11A1. Stringent analysis of this data with a FDR of less than 1% revealed 477 DEPs out of which 247 proteins were upregulated and 230 proteins were downregulated in A2780cis cells compared to A2780 cells (Supplementary Table 4). To identify major pathways that might contribute to COL11A1-mediated cisplatin resistance, we performed IPA on the proteins that were significantly upregulated in A2780cis cells. Interestingly, proteins of the FAO pathway were predominantly overexpressed in A2780cis cells (Fig. 1a, S1A) compared to A2780 cells. To investigate whether COL11A1 directly upregulates the expression of FAO enzymes in ES2, OVCAR3, and A2780 ovarian cancer cells that do not express COL11A1 or express very low endogenous levels of COL11A1^31^, we (i) co-cultured ES2 ovarian cancer cell line with scrambled control CAFs or COL11A1-knockdown CAFs; (ii) cultured ES2, OVCAR3 and A2780 ovarian cancer cell lines in plates coated with COL11A1 extracted from A204 cell line (COL11A1 extract); or (iii) supplemented ES2 cells with recombinant COL11A1 protein. We measured the expression levels of COL11A1 and FAO enzymes in ovarian cancer cells by RT-PCR and Western blotting. Culturing COL11A1-low cells in the presence of COL11A1 extract or CAFs did not induce COL11A1 expression in the cancer cells (Fig. S1C). The mRNA expression of FAO enzymes was increased in ES2 cells co-cultured with scrambled CAFs (Fig. 1B) or supplemented with recombinant COL11A1 protein (Fig. S1D). In addition, the protein expression of carnitine palmitoyl transferase 1 (CPT1A), the rate-limiting enzyme of FAO, was upregulated in ES2 (Fig. 1C, left), OVCAR3, and A2780 cells (Fig. 1C, right) cultured in COL11A1-coated plates. In contrast, knockdown of COL11A1 reduced the mRNA expression of FAO enzymes CPT1A, ACSL1, ACAA2, and HADHA in A2780cis cells (Fig. S1E, F). To further validate whether COL11A1 increases the FAO rate in ovarian cancer cells, we supplemented a long-chain fatty acid substrate, palmitate, to ES2 cells cultured on COL11A1 extract, and measured OCR using the Seahorse XF analyzer. ES2 cells grown on COL11A1-positive extract (A204-scrambled extract) showed increased OCR after palmitate addition (indicative of increased FAO rate) compared to ES2 cells grown on COL11A1-negative extract (A204-shCOL11A1 extract) (Fig. 1D, top). In contrast, knockdown of COL11A1 resulted in decreased FAO rate in A2780cis cells (Fig. S2D). COL11A1 also increased the levels of ATP and NADH, byproducts of FAO, in ES2 cells cultured on COL11A1 extract (Fig. 1E), which was attenuated by CPT1A knockdown using shRNAs (Fig. 1E, S1F-G) or treatment with a pharmacological inhibitor of CPT1A, etomoxir (Fig. S2A, B). Similarly, etomoxir treatment significantly reduced COL11A1-induced NADH levels in OVCAR3 cells (Fig. S2C), suggesting that COL11A1 increases ATP and NADPH production through FAO in ovarian cancer cells. Interestingly, the upregulation of FAO enzyme expression or FAO rate was not observed in ES2 cells cultured in type I collagen (COL1)-coated plates (Fig. 1C, D, bottom), implying that FAO upregulation might be specific to COL11A1. Of note, the levels of glycolysis, measured by ECAR after glucose addition, were not altered in ES2 cells cultured on COL11A1 extract (Fig. S2E) or A2780cis cells with or without COL11A1 expression (Fig. S2F). Taken together, these results suggest that COL11A1, but not type I collagen, upregulates the FAO process in ovarian cancer cells.

### COL11A1 upregulates FAO through the activation of DDR2/α1β1 integrin-Src-Akt-AMPK signaling in ovarian cancer

**Fig. 2:**
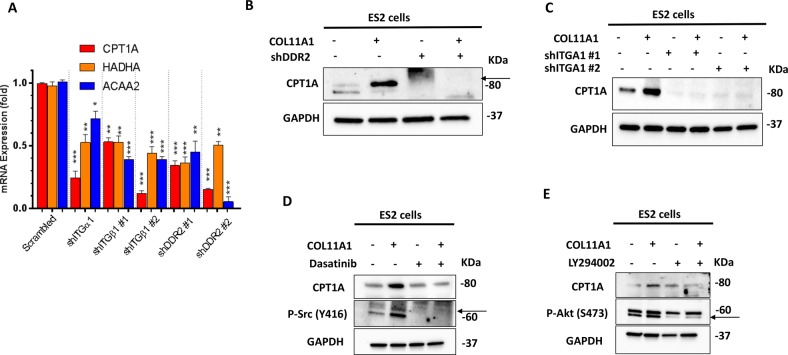
COL11A1 upregulates FAO through DDR2-α1β1 integrin-Src-Akt signaling. **a** Real-time PCR of CPT1A, ACAA2, and HADHA genes in ES2 cells expressing scrambled, shITGA1, shITGB1, or shDDR2. ES2 scrambled or receptor knockdown cells were cultured in COL11A1-coated plates for 72 h. The mRNA expression was normalized to RPL32. *N* = 3; *y*-axis, fold change; error bars, SD; *p < 0.05; **p < 0.01; ***p < 0.001. **b**, **c** Western blot of CPT1A in scrambled and shDDR2 (**b**) or shITGA1 (**c**) expressing ES2 cells. GAPDH was used as a loading control. *N* = 3. **d** Western blot of pSrc (Y416) and CPT1A in ES2 cells cultured in either PBS or COL11A1-coated plates for 48 h and treated with Dasatinib (2 µM) for 48 h. GAPDH was used as a loading control. *N* = 2. **e** Western blot of pAKT (S473) and CPT1A in ES2 cells cultured in either PBS or COL11A1-coated plates for 48 h and treated with LY294002 (20 µM) for 48 h. GAPDH was used as a loading control. *N* = 2.

We previously published that COL11A1 engages DDR2 receptor tyrosine kinase and α1β1 integrin on cell surface to transduce downstream signaling in ovarian cancer cells^31^. Therefore, we tested whether COL11A1 engages the same receptors to upregulate FAO in ovarian cancer cells. To address this question, we knocked down DDR2, ITGA1, and ITGB1 in ES2 cells using shRNAs (Fig. S3A–C), cultured them in COL11A1-coated plates and measured the expression levels of FAO enzymes. Knockdown of DDR2, ITGA1, or ITGB1 significantly decreased the expression levels of FAO enzymes in ES2 cells (Fig. 2A–C) and A2780 cells (Fig. S3D–G) despite the presence of COL11A1. This result suggests that COL11A1 engages DDR2 and α1β1 integrin to induce FAO in ovarian cancer cells.

Since we have shown that Src and Akt are activated by COL11A1 binding to DDR2 and α1β1 integrin^31^, we next determined whether activation of Src and Akt is necessary for the COL11A1-induced FAO upregulation in ovarian cancer cells. As expected, in ES2 cells, COL11A1 markedly increased the phosphorylation of Src and Akt (indicative of Src and Akt activation) as early as 2.5 h (Fig. S4A, C) which was retained even after 96 h of culture on COL11A1 (Fig. 2D, E). When we blocked Src activation using a pharmacological inhibitor dasatinib, it significantly reduced the expression of CPT1A even in the presence of COL11A1 in ES2, OVCAR3, and A2780 cells (Fig. 2d, S4D, E). Similarly, when we blocked Akt activation using pharmacological inhibitors LY294002 or MK2206, it significantly reduced the expression of CPT1A even in the presence of COL11A1 in ES2 and A2780 cells (Fig. 2e, S4F) and OVCAR3 cells (Fig. S4G). Collectively, these results suggest that COL11A1 activates Src and Akt to upregulate FAO in ovarian cancer cells.

**Fig. 3:**
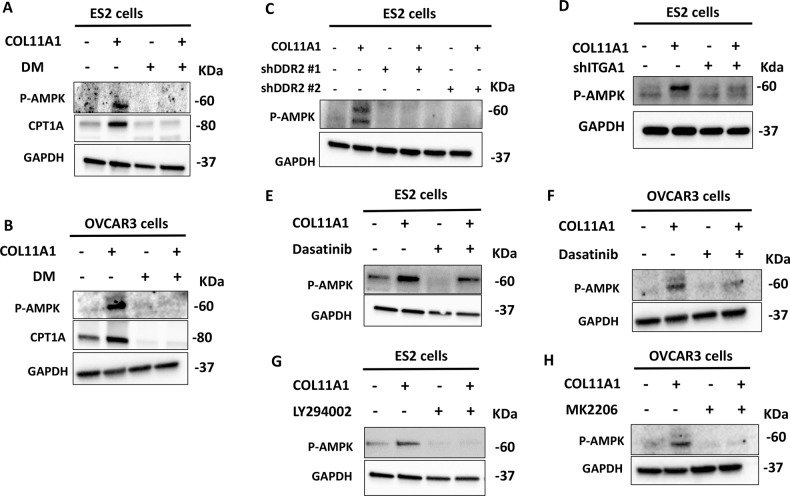
COL11A1 activates AMPK through DDR2-Src-Akt-dependent signaling. **a**, **b** Western blot of pAMPK (T172) and CPT1A in ES2 (**a**) or OVCAR3 (**b**) cells cultured in either PBS or COL11A1-coated plates for 48 h and treated with dorsomorphin (DM) at 8 µM for 48 h. GAPDH was used as a loading control. *N* = 2. **c**, **d** Western blot of pAMPK (T172) in ES2 cells expressing either scrambled or shDDR2 (**c**) and shITGA1 (**d**) cultured on either in PBS or COL11A1-coated plates for 48 h. GAPDH was used as a loading control. *N* = 2. **e**, **f** Western blot of pAMPK (T172) in ES2 (**e**) or OVCAR3 (**f**) cells cultured in either uncoated or COL11A1-coated plates for 48 h and treated with Dasatinib (2 µM for ES2 and 5 µM for OVCAR3) for 48 h. GAPDH was used as a loading control. *N* = 2. **g** Western blot of pAMPK (T172) in ES2 cells cultured in either PBS or COL11A1-coated plates for 48 h and treated with LY294002 (20 µM) for 48 h. GAPDH was used as a loading control. *N* = 2. **h** Western blot of pAMPK (T172) in OVCAR3 cells cultured in either PBS or COL11A1-coated plates for 48 h and treated with MK2206 (20 µM) for 48 h. GAPDH was used as a loading control. *N* = 2.

5′ AMP-activated protein kinase (AMPK) plays a central role in maintaining energy homeostasis by upregulating FAO^38^. Therefore, we questioned whether AMPK is activated by COL11A1 to upregulate FAO in ovarian cancer cells. We demonstrated that phosphorylation of AMPK (indicative of AMPK activation) was increased by COL11A1 as early as 2.5 h and was retained until 96 h in ovarian cancer cells. (Fig. 3a–h, S3B). In addition, using a Human Protein Kinase Array, we demonstrated that AMPK was one of the phosphoproteins upregulated in response to culturing ovarian cancer cells on COL11A1. (Fig. S4H). Treating ES2 and OVCAR3 cells with an AMPK inhibitor dorsomorphin (DM) abrogated COL11A1-dependent increase in AMPK phosphorylation and CPT1A expression (Fig. 3a, b). Furthermore, knocking down AMPK expression in ES2 cells using shRNAs also resulted in loss of FAO enzyme expression (Fig. S4I) suggesting that AMPK acts as an upstream regulator of FAO.

We next questioned whether DDR2-α1β1 integrin-Src-Akt signaling is important for COL11A1-induced AMPK activation. Knockdown of DDR2 dramatically abrogated COL11A1-induced AMPK phosphorylation (Fig. 3c), while knockdown of ITGA1 yielded a moderate reduction in AMPK phosphorylation (Fig. 3d), suggesting that DDR2 might be the predominant receptor that mediates COL11A1-induced AMPK activation. Treatment of ES2 and OVCAR3 cells with Src and Akt inhibitors also significantly attenuated AMPK phosphorylation despite the presence of COL11A1 (Fig. 3e–h). Taken together, these results suggest that AMPK acts as a downstream effector of COL11A1-DDR2-Src-Akt signaling to upregulate FAO in ovarian cancer cells.

### COL11A1-mediated FAO upregulation is due to an increase in de novo fatty acid synthesis

Next, we investigated the source of fatty acids utilized for FAO. It has been shown that ovarian cancer cells uptake exogenous fatty acids from the omentum to acquire tumorigenic and metastatic phenotype^24,27^. Therefore, we asked if COL11A1 also increases the uptake of extracellular fatty acids to promote FAO in ovarian cancer cells. We blocked CD36 (a long-chain fatty acid transporter) using blocking antibodies and measured the OCR after supplementation of exogenous palmitate (indicative of FAO rate). However, CD36 blocking antibodies only slightly decreased the FAO rate in ES2 and OVCAR3 cells cultured in COL11A1-coated plates (Fig. S5A, B), suggesting that exogenous fatty acids might not be a major source of fatty acids for FAO.

**Fig. 4:**
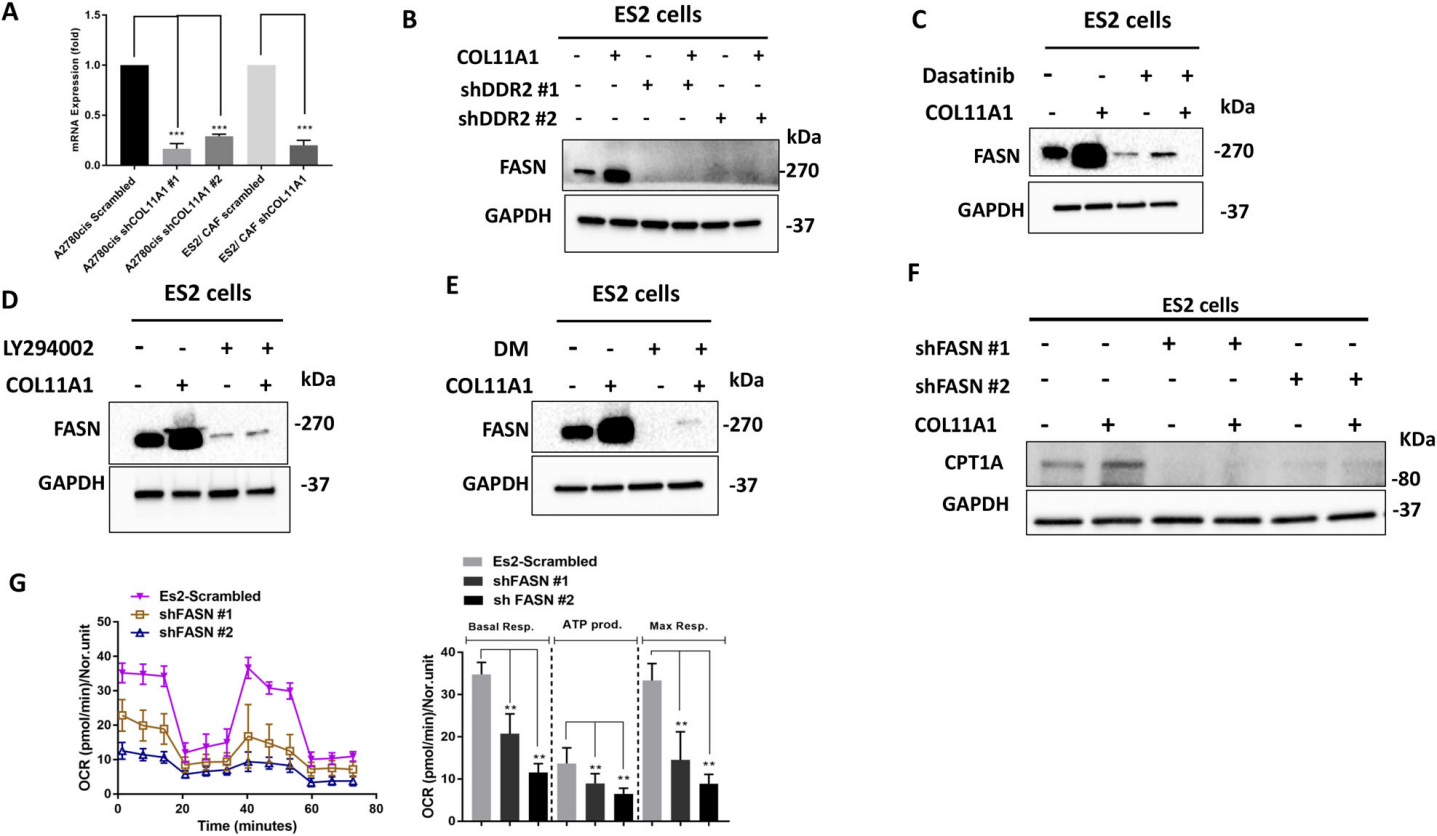
COL11A1 upregulates fatty acid synthesis in ovarian cancer cells. **a** Real-time PCR of FASN in A2780cis-scrambled and A2780cis-shCOL11A1 and in ES2 cells co-cultured with CAF-scrambled or CAF-shCOL11A1. The mRNA expression of FASN was normalized to RPL32. *N* = 3; *y*-axis, fold change; error bar, SD; ***p < 0.001. **b** Western blot of FASN in ES2 cells expressing scrambled and DDR2 shRNA. GAPDH was used as a loading control. *N* = 2. **c** Western blot of FASN in ES2 cells cultured in either PBS or COL11A1-coated plates for 48 h and treated with Dasatinib (2 µM) for 48 h. GAPDH was used as a loading control. *N* = 2. **d** Western blot of FASN in ES2 cells cultured in either PBS or COL11A1-coated plates for 48 h and treated with LY294002 (20 µM) for 48 h. GAPDH was used as a loading control. *N* = 2. **e** Western blot of FASN in ES2 cells cultured in either PBS or COL11A1-coated plates for 48 h and treated with DM (8 µM) for 48 h. GAPDH was used as a loading control. *N* = 2. **f** Western blot of CPT1A in ES2 scrambled and shFASN cells cultured in either PBS or COL11A1-coated plates. GAPDH was used as a loading control. *N* = 2. **g** A real-time oxygen consumption rate (OCR) in response to sequential treatments with palmitate, oligomycin, FCCP, and antimycin-A/rotenone in ES2 scrambled and shFASN cells cultured in XFp plates that are coated with COL11A1. Quantification of the OCR is shown on the right. *N* = 3; *y*-axis, OCR; error bar, SD; **p < 0.01.

Cancer cells increase de novo fatty acid synthesis to provide fatty acid pools to generate essential structural and signaling components and meet their high metabolic demands^8^. Therefore, we hypothesized that COL11A1 might upregulate fatty acid synthesis to supply cancer cells with fatty acids to promote FAO. Indeed, we observed an increased expression of FASN in ES2 cells co-cultured with COL11A1-expressing CAFs (Fig. 4A) or supplemented with recombinant COL11A1 (Fig. S1D) or cultured in COL11A1-coated plates (Fig. 4b–f), and in OVCAR3 and A2780 cells cultured in COL11A1-coated plates (Fig. S5C–F). Interestingly, FASN expression was not altered in ES2 cells cultured on type I collagen (data not shown). On the other hand, FASN expression was reduced by COL11A1 knockdown in A2780cis cells (Fig. 4A). Interestingly, DDR2 knockdown significantly abrogated COL11A1-induced FASN overexpression (Fig. 4B) while ITGA1 knockdown only slightly reduced FASN expression (data not shown), suggesting that DDR2 might be the predominant receptor that mediates COL11A1-induced FASN expression in ovarian cancer cells.

To determine whether the effectors of the COL11A1 signaling, Src, Akt, and AMPK, also play a role in upregulating FASN in ovarian cancer cells, we blocked these signaling molecules using pharmacological inhibitors and measured the expression of FASN. Inhibitors of Src (dasatinib), Akt (LY294002), and AMPK (DM) significantly reduced FASN protein expression even in the presence of COL11A1 in ES2 (Fig. 4c–e) and OVCAR3 cells (Fig. S5C–E). FASN mRNA expression was also reduced upon LY294002 treatment in A2780 cells (Fig. S5F).

To test whether fatty acid synthesis modulates FAO, we knocked down FASN in ES2 cells (Fig. S6A), cultured them in uncoated or COL11A1-coated plates and measured CPT1A expression and OCR. We observed a dramatic decrease in CPT1A expression and FAO rate in FASN-knockdown ES2 cells (Fig. 4F, G), suggesting that fatty acid synthesis might be an upstream regulator of FAO in ovarian cancer cells. Similarly, OVCAR3 cells treated with C75, a competitive, irreversible FASN inhibitor, showed a marked reduction in CPT1A expression despite culturing on COL11A1 (Fig. S6B). Collectively, our results demonstrate that COL11A1 increases de novo fatty acid synthesis through DDR2-Src-Akt-AMPK signaling to upregulate FAO in ovarian cancer cells.

### Inhibition of FAO restores sensitivity of ovarian cancer cells to cisplatin

**Fig. 5:**
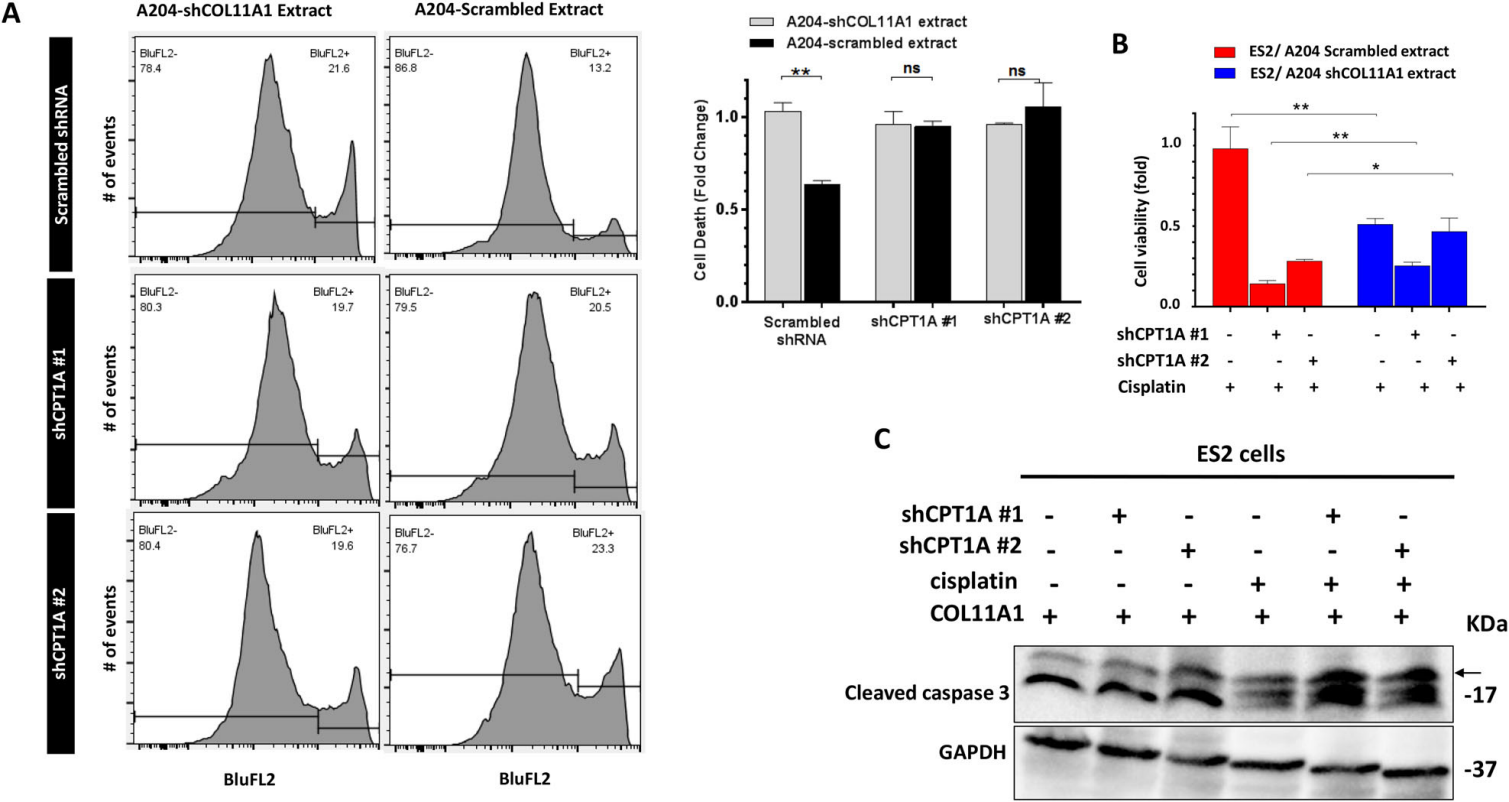
Inhibition of FAO attenuates COL11A1-induced cisplatin resistance in ovarian cancer cells. **a** Flow cytometry analysis of propidium iodide (PI) staining in ES2 cells expressing either scrambled or shCPT1A cultured in COL11A1-positive or negative extract for 48 h and treated for cisplatin (16 µM) for 72 h. Representative flow peak charts are shown on left and quantification is shown on the right. *N* = 3; *y*-axis, cell death (fold change); error bar, SD; ns not significant; **p < 0.01. **b** Relative cell viability of ES2 cells expressing scrambled or shCPT1A vectors cultured in either COL11A1-positive or COL11A1-negative extract and treated with cisplatin (16 µM) for 72 h. *N* = 3; *y*-axis, cell viability (fold change); Error bar, SD; *p < 0.05; **p < 0.01. **c** Western blot of cleaved caspase 3 in ES2 cells expressing scrambled or shCPT1A treated with cisplatin (16 µM) cultured in COL11A1-coated plates for 48 h. GAPDH was used as a loading control.

Given our initial observation that FAO is upregulated in cisplatin-resistant variant of A2780 cell line (A2780cis), we sought to test whether FAO inhibition can attenuate COL11A1-induced cisplatin resistance in ovarian cancer cells. For this, we knocked down CPT1A in ES2 cells, cultured them on COL11A1 extract, and measured cell viability and apoptosis after cisplatin treatment. An appropriate dose of cisplatin was chosen after performing a dose response experiment in scrambled and shCPT1A cells (Fig. S6C). ES2 cells cultured on COL11A1-positive extract exhibited a significant decrease in cell death upon cisplatin treatment, compared to ES2 cells cultured on COL11A1-negative extract (Fig. 5a; 13.2% vs. 21.6% cell death, *p* < 0.01). However, CPT1A-knockdown ES2 cells grown on COL11A1-positive extract exhibited similar percentage of cell death as CPT1A-knockdown ES2 cells on COL11A1-negative extract (Fig. 5a, b). Furthermore, CPT1A knockdown in ES2 cells significantly increased apoptosis (measured by cleaved caspase 3) upon cisplatin treatment despite the presence of COL11A1 (Fig. 5c; data not shown). Etomoxir treatment in ES2 cells also resulted in a significant decrease in mitochondrial membrane potential (measured by TMRE staining; Fig. S5F), an early event in apoptosis. Notably, FASN knockdown also resulted in decreased TMRE intensity despite the presence of COL11A1, further suggesting that fatty acid synthesis increases mitochondrial activity via FAO (Fig. S5F). Collectively, our results suggest that COL11A1 inhibits cisplatin-induced apoptosis by upregulating FAO in ovarian cancer cells.

### FAO upregulation is a marker for poor prognosis in ovarian cancer patients

**Fig. 6:**
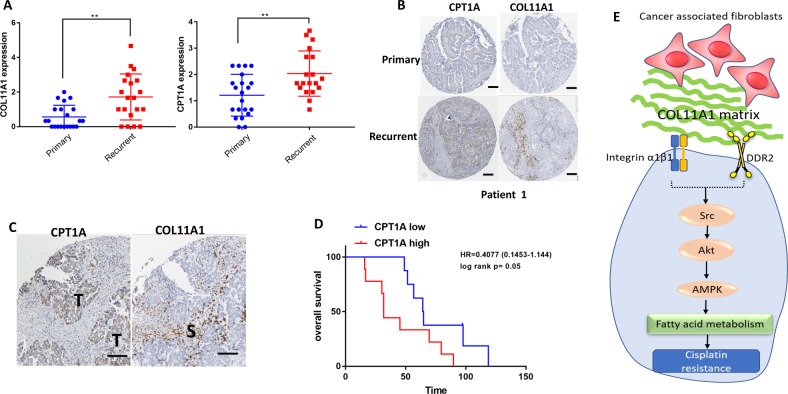
FAO upregulation is associated with poor prognosis in ovarian cancer patients. **a** Protein expression of COL11A1 (left) and CPT1A (right) in the matched primary and recurrent ovarian tumors. *N* = 21. *y*-Axis, protein expression; **p < 0.01. **b** Representative images of tumor-specific CPT1A and stroma-specific COL11A1 staining in matched primary and recurrent ovarian tumors. Scale bar 200 µm. **c** Representative images of staining of CPT1A in tumor cells adjacent to COL11A1-positive stroma in matched recurrent ovarian tumors. S stroma, T tumor. Scale bar 100 µm. **d** Overall survival in patients with recurrent tumors expressing high levels of CPT1A compared to primary tumors vs. patients with recurrent tumors showing no increase in CPT1A expression compared to primary tumors. *, log rank *p* < 0.05. **e** A proposed mechanism underlying COL11A1-induced cisplatin resistance in ovarian cancer.

To determine whether FAO genes are upregulated in human ovarian cancer tissues, we searched publicly available human protein atlas data for ovarian cancer. We found that CPT1A is upregulated in ovarian tumors compared to normal ovaries (Fig. S7A). To determine whether high levels of FAO genes are associated with poor prognosis, we correlated the expression levels of FAO genes (CPT1A, ACAA2, and HADHA) with overall survival in high-grade serous ovarian cancer patients using the Kaplan–Meier plotter (https://kmplot.com). We discovered that high levels of FAO gene expression are associated with poor overall survival in 1207 high-grade serous ovarian cancer patients (Fig. S7B). Next, we used TMA that contained 21 patient-matched primary, metastatic and recurrent ovarian tumor samples to correlate the expression levels of FAO enzymes with clinical outcome (Supplementary Table 5). Staining of TMA showed an increase in CPT1A expression in recurrent tumors compared to primary tumors (Fig. S7D). Similarly, the TMA also showed increased COL11A1 expression in recurrent tumors compared to primary tumors (Fig. S7D). In recurrent ovarian tumors, CPT1A expression was positively correlated with COL11A1 expression (Fig. S7C). Representative images showing matched primary and recurrent tumors with increased CPT1A and COL11A1 expression are shown in Fig. 6b and Fig. S7D. Further analysis of TMA revealed that tumor epithelial cells adjacent to COL11A1-postive stroma showed the strongest CPT1A expression (Fig. 6C, Fig. S7E). Patients who showed increased CPT1A expression in recurrent tumors compared to primary tumors, exhibited poor overall survival compared to patients whose tumors did not show an increase in CPT1A expression in recurrent vs. primary tumors (Fig. 6d). These data suggest that high levels of FAO are associated with poor prognosis in ovarian cancer.

## Discussion

Emerging evidence suggests that tumor cells exhibit metabolic flexibility to develop therapy resistance^39^, although the underlying mechanisms are poorly understood. Here, we report that the extracellular matrix (ECM) proteins in the tumor microenvironment can alter the metabolic phenotype of ovarian cancer cells to confer chemotherapy resistance. In particular, we showed that COL11A1, a collagen subtype specifically overexpressed in cisplatin-resistant ovarian cancer^25–27,30^, upregulates FAO to promote cisplatin resistance in ovarian cancer cells. Mechanistically, we demonstrate that COL11A1 predominantly binds to DDR2 and activates Src-Akt-AMPK signaling to induce FAO (Fig. 6e) and blocking FAO attenuates COL11A1-induced cisplatin resistance by increasing mitochondrial apoptosis pathway. Furthermore, we discovered that COL11A1 upregulates de novo fatty acid synthesis to derive fatty acids for oxidation in ovarian cancer cells. In patient tumors, FAO is increased concurrently with COL11A1 and is associated with poor survival in recurrent ovarian cancer.

The role of fatty acids as substrates that induce chemoresistance is less explored compared to glucose or glutamine^40^. In this study, we showed that cisplatin-resistant ovarian cancer cells preferentially upregulate FAO and rely on fatty acids for their survival and the inhibition of FAO sensitizes them to cisplatin. Our work is further supported by the findings of others that chemoresistant ovarian cancers exhibit a high metabolically active phenotype with the ability to utilize fatty acids as fuel^41,42^. FAO upregulation increases both mitochondrial function and cellular pools of NADH and ATP (Fig. 1e, S6D). ATP and NADH are known to provide a survival advantage to cancer cells^19^ and thus might contribute to cisplatin resistance as well. In concordance with our study, FAO inhibition has been shown to cause chemosensitization by limiting ATP supply in prostate cancer^43^ and leukemia cells^44^.

The interplay between the ECM and cancer cells can regulate a plethora of cellular functions including cellular proliferation, differentiation, metabolism, and drug resistance^45,46^. Several collagens have been associated with cancer progression and chemoresistance, including types I, III, V, VI, and XI^27–29,33,47^, however, their role in regulating cancer cell metabolism has not been explored. Here, for the first time, we identified that type XI collagen in the tumor microenvironment can switch the metabolic preferences of ovarian cancer cells toward FAO. While type I collagen has been shown to upregulate glycolysis^48^, our data show that type I collagen does not upregulate FAO in ovarian cancer cells. In contrast, COL11A1 does not change glycolysis, yet preferentially upregulates fatty acid metabolism to promote cisplatin resistance in ovarian cancer cells. It remains to be determined whether the differences in metabolic preferences induced by different collagen subtype arises from the binding to different cell surface receptors and/or activation of different downstream signaling components. We identified that DDR2 might be a predominant receptor for COL11A1 to activate downstream signaling to upregulate fatty acid metabolism. Whether DDR2 also plays a role in type I collagen-mediated signaling and to what extent needs to be studied. To develop more effective therapy to block chemoresistance, it is important to understand which collagen subtypes are enriched in the tumor microenvironment at specific disease stages and how these collagen subtypes dictate specific bioenergetic phenotypes of cancer cells to confer chemoresistance.

Our study showed that a blockade of CD36, a fatty acid transporter known to drive FAO^37^, did not affect FAO in the presence of COL11A1, suggesting that CD36-mediated fatty acid uptake only minimally supports FAO in ovarian cancer cells that reside in the COL11A1-rich microenvironment. Although this observation is somewhat in contrast with other studies showing a positive regulatory role of extracellular fatty acids on FAO^23,24^, it is unclear at this point whether other fatty acid transporters might alter flux of fatty acids into the cells and promote FAO in the presence of COL11A1. Nevertheless, it is interesting that ovarian cancer cells can upregulate both synthesis and oxidation of fatty acids in the presence of COL11A1. Given that fatty acid synthesis and oxidation can negatively regulate each other, this poses an intriguing question as to how the ovarian cancer cells are able to overcome the regulatory effects of malonyl CoA, a substrate for fatty acid synthesis and an allosteric inhibitor of CPT1A. Although coupling of fatty acid synthesis with FAO seems to be bioenergetically unfavorable, this phenomenon has been documented in adipose tissue, muscle, and T cells^49–51^. Furthermore, co-suppression of FAO and fatty acid synthesis showed synergistic effects in killing prostate cancer cells and myeloma cells and blocking metastasis of ovarian cancer cells^23,52,53^, suggesting co-activation of these pathways. There are two possible explanations for co-activation of FAO and fatty acid synthesis. Firstly, ovarian cancer cells might oscillate between periods of FAO and fatty acid synthesis activation. Secondly, malonyl COA might be compartmentalized to prevent inhibition of CPT1A so that FAO is uninterrupted or rapidly utilized to meet cancer cells’ high demand for energy and/or biomolecules.

In summary, we identified that COL11A1 increases FAO in ovarian cancer cells to confer cisplatin resistance and COL11A1 and FAO are concurrently upregulated in recurrent ovarian tumors. FAO inhibitors, such as etomoxir, perhexiline, and ranolazine, have been approved by FDA and/or EMA for heart diseases and are currently being evaluated in various stages of clinical trials as anticancer agents^54–57^. Therefore, targeting FAO using these drugs might be a promising therapeutic strategy for cisplatin-resistant recurrent ovarian cancer, which often overexpress COL11A1.

## Supplementary information

Supplemental materials and figure legends

Supplemental figure 1

Supplemental figure 2

Supplemental figure 3

Supplemental figure 4

Supplemental figure 5

Supplemental figure 6

Supplemental figure 7

Supplemental table 4
