# Supplementary material for: Inhibition of collagen XI alpha 1-induced fatty acid oxidation triggers apoptotic cell death in cisplatin-resistant ovarian cancer
Source: Cell Death Dis. 2020 Apr 20;11(4):258. doi: 10.1038/s41419-020-2442-z (PMC7171147; doi:10.1038/s41419-020-2442-z)
Supplement: Supplementary file 1 — Supplemental materials and figure legends [file 41419_2020_2442_MOESM1_ESM.docx]

**Supplementary Information**

**Supplementary Table 1: Plasmid constructs**

| **Plasmid construct/ mission shRNA plasmid DNA** | **Catalog #** | **Source** |
| --- | --- | --- |
| pCMV-ΔR 8.2 dvpr | Addgene #8455 | Gift from Dr. Bob Weinberg |
| pCMV-VSVG | Addgene #8454 | Gift from Dr. Bob Weinberg |
| scrambled | #SHC016 | Sigma-Aldrich |
| shCOL11A1 #1 and #2 | #TRCN0000083377  #TRCN0000433589 | Sigma-Aldrich |
| shDDR2 #1 and #2 | #TRCN0000121262  #TRCN0000196465 | Sigma-Aldrich |
| shITGα1 #1 and #2 | #TRCN0000057752  #TRCN0000057748 | Sigma-Aldrich |
| shITGβ1 #1 and #2 | #TRCN0000122922  #TRCN0000122921 | Sigma-Aldrich |
| shCPT1A #1 and #2 | #TRCN0000036279  #TRCN0000036283 | Sigma-Aldrich |
| shFASN #1, #2, #3, #4 and #5 | \| #TRCN0000003125 \| \| --- \| \| #TRCN0000003126 \| \| #TRCN0000003127 \| \| #TRCN0000003128 \| \| #TRCN0000003129 \| | Sigma-Aldrich |
| shAMPK #1 and #2 | #TRCN0000000858  #TRCN0000000860 | Sigma-Aldrich |

**Supplementary Table 2: Antibodies and inhibitors**

| **Antibody/ inhibitor** | **Catalog #** | **Source** |
| --- | --- | --- |
| Anti-ITGα1 | #sc-10728 | Santa Cruz Biotechnology |
| Anti-ITGβ1 | #MAB1959 | EMD Millipore |
| Anti-cleaved caspase | #9664P | Cell Signaling Technology |
| Anti-CPT1A | #AB128568 | Abcam |
| Anti-HADHA | #ab54477 | Abcam |
| Anti-ACAA2 | #ab128929 | Abcam |
| Anti-p-Src (Y416) | #2101 | Cell signaling Technology |
| Anti-p-AMPK (T172) | #2535S | Cell signaling Technology |
| Anti-FASN | #AB22759 | Abcam |
| Anti-COL11A1 | #ab64883 | Abcam |
| Anti-DDR2 | #Y740 | RD Systems |
| Anti-GAPDH | #10R-G109a | Fitzgerald |
| Anti-CD36 (blocking) | AB23680 | Abcam |
| Anti- pAkt (S473) | #44623G | Life technologies |
| Goat-anti mouse secondary Ab  Goat-anti rabbit secondary Ab | #1706516  #1721019 | Bio-Rad |
| Donkey-anti goat secondary Ab | #AB97110 | Abcam |
| Recombinant COL11A1 | CSB-EP005716H4 (A4) | Lifeome Biolabs |
| Type I collagen | #5005 | Advanced BioMatrix |
| Cisplatin | #P4394 | Sigma Aldrich |
| LY294002 | #tlrl-ly29 | InvivoGen |
| Dasatinib | #73082 | Stem Cell technologies |
| Dorsomorphin | #AB144821 | Abcam |
| MK2206 | #A3010 | ApexBio |
| TMRE | #AB113852 | Abcam |
| C75 | #45-C5490-5MG | Sigma Aldrich |

**Supplementary Table 3: Primer information**

| Gene | Primer sequence |
| --- | --- |
| RPL32 | F: 5’-ACAAAGCACATGCTGCCCAGTG-3’  R: 5’-TTCCACGAT-GGCTTTGCGGTTC-3’ |
| CPT1A | F: 5’- TCCAGTTGGCTTATCGTGGTG-3’  R: 5’- CTAACGAGGGGTCGATCTTGG-3’ |
| HADHA | F: 5’-GAGGTTGCCATTTCATGCCA-3’  R: 5’- ACCAGTCAGCATCATGTCCA-3’ |
| ACSL1 | F: 5’-CTTCTGGTACGCCACGAGAC-3’  R: 5’- GTCGCTGTCAAGTAGTGCG-3’ |
| ACAA2 | F: 5’-GGCACTGAAGAAAGCAGGACTG-3’  R: 5’-GTGACCCAAAGCAATGGCTCCT-3’ |
| FASN | F: 5’-CTTCCGAGATTCCATCCTACGC-3’  R: 5’-TGGCAGTCAGGCTCACAAACG-3’ |
| AMPK | F: 5’ -AGGAAGAATCCTGTGACAAGCAC-3’  R: 5’-CCGATCTCTGTGGAGTAGCAGT-3’ |

**Supplementary Table 4: A list of 477 differentially expressed proteins between A2780cis cells and A2780 cells.**

**Supplementary table 5**

**Clinical characteristics of 21 patient tumors used in TMA sections**

| Characteristics | total | number/percentage | | | |  |
| --- | --- | --- | --- | --- | --- | --- |
|  |  |  |  | |  | |
| **Age (years)** |  |  |  | |  | |
|  |  |  |  | |  | |
| <60 | 21 | 14 (66.6%) | |  | |  |
| >60 | 21 | 7 (33.3%) |  | |  | |
|  |  |  |  | |  | |
| **Stage** |  |  |  | |  | |
| I | 21 | 0 (0%) |  | |  | |
| II | 21 | 1 (5%) |  | |  | |
| III | 21 | 16 (76%) |  | |  | |
| IV | 21 | 4 (19%) |  | |  | |
|  |  |  |  | |  | |
| **Grade** |  |  |  | |  | |
| 1 and 2 | 21 | 1 (5%) |  | |  | |
| 3 | 21 | 20 (95%) |  | |  | |
|  |  |  |  | |  | |
| **Histology** |  |  |  | |  | |
| Papillary serous | 21 | 21 (100%) |  | |  | |
| others | 21 | 0 (0%) |  | |  | |
|  |  |  |  | |  | |
| **Months to relapse** |  |  |  | |  | |
| <20 | 21 | 14 (66.6%) | |  | |  |
| 21-50 | 21 | 5 (23.8%) |  | |  | |
| >50 | 21 | 1 (4.7%) |  | |  | |
| NA | 21 | 1 (4.7%) |  | |  | |
|  |  |  |  | |  | |
| **Survival (months)** |  |  |  | |  | |
| <20 | 21 | 2 (9.5%) |  | |  | |
| 21-80 | 21 | 12 (57.1%) | |  | |  |
| >80 | 21 | 7 (33.3%) |  | |  | |

**Supplementary figure legends**

**Figure S1. COL11A1 upregulates FAO in ovarian cancer cells. (a)** Western blot analysis of FAO enzymes CPT1A, HADHA, and ACAA in A2780 cells compared to A2780cis cells. N=2. GAPDH was used as a loading control. **(b)** Western blot of COL11A1 in A204 scrambled or shCOL11A1 cells. GAPDH was used as a loading control. **(c)** Western blot of COL11A1 in ES2 cells cultured on A204 scrambled or shCOL11A1 extract. GAPDH was used as a loading control. **(d)** Real-Time PCR of FA metabolism genes in ES2 cells supplemented with recombinant COL11A1 protein. N=3, Y axis, mRNA expression (fold change); error bars SD; ***, p<0.001. The mRNA expression was normalized to RPL32. **(e)** Western blot of COL11A1 in A2780cis scrambled and shCOL11A1 cells. GAPDH was used as a loading control. **(f)** Real-Time PCR of FAO genes in A2780cis cells with or without COL11A1 shRNA. N=3, Y axis, mRNA expression (fold change); error bars SD; ***, p<0.001. The mRNA expression was normalized to RPL32. **(g)** Western blot of CPT1A in ES2 scrambled or shCPT1A cells. GAPDH was used as a loading control. **(h)** OCR in response to sequential treatments with palmitate, oligomycin, FCCP, and antimycin-A/rotenone in ES2 scrambled or shCPT1A cells cultured in XFp plates coated with COL11A1 (left). Quantification of the OCR is shown on the right. N=3; Y axis, OCR; Error bars, SD; **, p<0.01.

**Figure S2. COL11A1 upregulates FAO but not glycolysis in ovarian cancer cells. (a)** Relative ATP levels in ES2 cells cultured in either COL11A1-positive or COL11A1-negative extract and treated with 40µM etomoxir for 1h. N=3; Y axis, relative ATP levels (fold change); error bars, SD; ns, not significant; ***, p<0.001.**(b)** Relative NADH levels in ES2 cells cultured in either COL11A1-positive or COL11A1-negative extract and treated with 40µM etomoxir for 1h. N=3; Y axis, relative NADH levels (fold change); error bars, SD; ns, not significant; *, p<0.05. **(c)** Relative NADH levels in OVCAR3 cells cultured in either COL11A1 or PBS coated plates and treated with 40µM etomoxir for 1h. N=3; Y axis, relative NADH levels (fold change); error bars, SD; *, p<0.05, **, p<0.01. **(d)** OCR in response to sequential treatments with palmitate, oligomycin, FCCP, and antimycin-A/rotenone in A2780cis scrambled or shCOL11A1 cells (top). Quantification of the OCR is showed on the bottom. N=3; Y axis, OCR; Error bars, SD; ns, not significant; *, p<0.05; ***, p<0.001. **(e)** ECAR in response to sequential treatments with glucose, oligomycin and 2-DG in ES2 cells cultured in either COL11A1 positive or COL11A1 negative extract. Quantification of the ECAR is shown on the bottom. N=3; Y axis, fold change; Error bar, SD; ns, not significant. **(f)** ECAR in response to sequential treatments with glucose, oligomycin and 2-DG in A2780cis scrambled and shCOL11A1 cells. Quantification of the ECAR is shown on the bottom. N=3; Y axis, fold change; Error bars, SD; ns, not significant.

**Figure S3. COL11A1 upregulates FAO in an α1β1 integrin and DDR2-dependent manner. (a)** Western blot of ITGA1 in ES2 scrambled or shITGA1 cells cultured in PBS or COL11A1-coated plates. GAPDH was used as a loading control. N=2. **(b)** Western blot of DDR2 in ES2 scrambled or shDDR2 cells cultured in PBS or COL11A1-coated plates. GAPDH was used as a loading control. N=2. **(c)** Western blot of ITGB1 in ES2 scrambled or shITGB1 cells. GAPDH was used as a loading control. **(d)** Western blot of DDR2 in A2780 scrambled or shDDR2 cells cultured in PBS or COL11A1-coated plates. GAPDH was used as a loading control. **(e)** Western blot of ITGA1 in A2780 cells with scrambled or shITGA1 expression cultured in PBS or COL11A1-coated plates. GAPDH was used as a loading control. **(f-g)** Real-time qPCR of CPT1A, HADHA, ACAA2 and FASN in A2780 cells with shITGA1 (f) or shDDR2 (g) expression. The mRNA expression was normalized to RPL32. N=3; Y axis, fold change; Error bars, SD; *, p<0.05; **, p<0.01.

**Figure S4. COL11A1 upregulates FAO in a Src-Akt-dependent manner. (a-c)** Western blot of p-Src (a), p-AMPK (b) and p-Akt (c) in ES2 cultured in COL11A1 for different time points; 2.5, 5, 8, 48 h for p-Src and p-AMPK and 2.5, 8 and 48h for p-Akt. GAPDH was used as a loading control **(d)** Western blot of CPT1A in OVCAR3 cells cultured in PBS or COL11A1-coated plates for 48h and treated with 5µM Dasatinib for 48h. GAPDH was used as a loading control. N=2 **(e-f)** Real time qPCR of CPT1A in A2780 cells cultured in PBS or COL11A1-coated plates and treated with 5µM Dasatinib (e) or 20 µM LY294002 (f) for 48h. The mRNA expression was normalized against RPL32. N=3; Y axis, fold change; Error bar, SD; *, p<0.05; **, p<0.01, ***, p<0.001. **(g)** Western blot of CPT1A in OVCAR3 cells cultured in PBS or COL11A1-coated plates for 48h and treated with 20µM MK2206 for 48h. GAPDH was used as a loading control. N=2. **(h)** Protein kinase array dot density analysis of pAMPK (T183) in ES2 cells cultured in either PBS or COL11A1-coated plates for 72h. N=2. **(i)** Real-time qPCR of AMPK, CPT1A, HADHA, ACAA2 and FASN in ES2 cells expressing scrambled or shAMPK. The mRNA expression was normalized to RPL32. N=3; Y axis, fold change; Error bars, SD; ***, p<0.01.

**Figure S5. COL11A1 upregulates fatty acid synthesis in ovarian cancer cells. (a-b)** OCR in response to sequential treatments with palmitate, oligomycin, FCCP, and antimycin-A/rotenone in ES2 cells (a) or OVCAR3 (b) cultured in PBS or COL11A1-coated plates and incubated with the IgA isotype control or anti-CD36 antibody overnight. Quantification of the OCR is showed on the right. N=3; Y axis, OCR; Error bars, SD; ns, not significant; **, p<0.01. **(c)** Western blot of FASN in OVCAR3 cells cultured in PBS or COL11A1-coated plates for 48h and treated with 5µM Dasatinib for 48h. GAPDH was used as a loading control. N=2. **(d)** Western blot of FASN in OVCAR3 cells cultured in either PBS or COL11A1-coated plates for 48h and treated with MK2206 (20µM) for 48h. GAPDH was used as a loading control. N=2 **(e)** Western blot of FASN in OVCAR3 cells cultured in either PBS or COL11A1-coated plates for 48h and treated with DM (8µM) for 48h. GAPDH was used as a loading control. N=2. **(f)** Real-time qPCR of FASN in A2780 cells cultured in PBS or COL11A1-coated plates for 48h and treated with 20µM LY294002 for 48h. The mRNA expression was normalized against RPL32. N=3; Y axis, fold change; Error bar, SD; ns, not significant; *, p<0.05; **, p<0.01, ***, p<0.001.

**Figure S6. Fatty acid synthesis drives FAO in ovarian cancer cells. (a)** Western blot of FASN in ES2 expressing scrambled shRNA or shFASN. GAPDH was used as a loading control. **(b)** Real-time qPCR of FASN and CPT1A in OVCAR3 cells cultured in PBS or COL11A1-coated plates for 48h and treated with 20µM C75 for 48h. The mRNA expression was normalized against RPL32. N=3; Y axis, fold change; Error bar, SD; ***, p<0.001. **(c)** Cell viability (fold) of ES2 scrambled and shCPT1A cells treated with different doses of cisplatin (4, 8, 16µM) for 72h. Y axis, fold change; Error bar, SD. **(d)** TMRE intensity in ES2 cells treated with etomoxir (40µM), or ES2 shFASN cells, cultured in COL11A1 extract. FCCP-treated samples serve as a negative control. Quantification is shown on the right. N=2; Y axis, fold change; Error bar, SD; **, p<0.01.

**Figure S7. FAO upregulation is associated with poor prognosis in ovarian cancer patients. (a)** Representative images of CPT1A staining in normal (n=3) and cancerous ovarian tissue (n=9). Scale bar 200 µm. **(b)** Kaplan-Meier survival curve plotted for 1,207 ovarian cancer patients with low or high mean expression of mitochondrial fatty acid oxidation proteins. Y axis, probability of survival; **, log rank p =0.0034. **(c)** Pearson correlation between COL11A1 expression (*in situ* hybridization) and CPT1A expression (immunostaining) in the matched primary and recurrent ovarian cancer. N=21; R=0.6; p<0.05. **(d)** Representative images of tumor-specific CPT1A and stroma-specific COL11A1 staining in matched primary and recurrent ovarian tumors. Scale bar 200 µm. **(e)** Representative images of CPT1A staining in tumor cells adjacent to COL11A1-positive stroma in matched recurrent ovarian tumors. S, stroma; T, tumor. Scale bar 100 µm.
