# Supplementary figures and images for: Inhibition of collagen XI alpha 1-induced fatty acid oxidation triggers apoptotic cell death in cisplatin-resistant ovarian cancer
Source: Cell Death Dis. 2020 Apr 20;11(4):258. doi: 10.1038/s41419-020-2442-z (PMC7171147; doi:10.1038/s41419-020-2442-z)

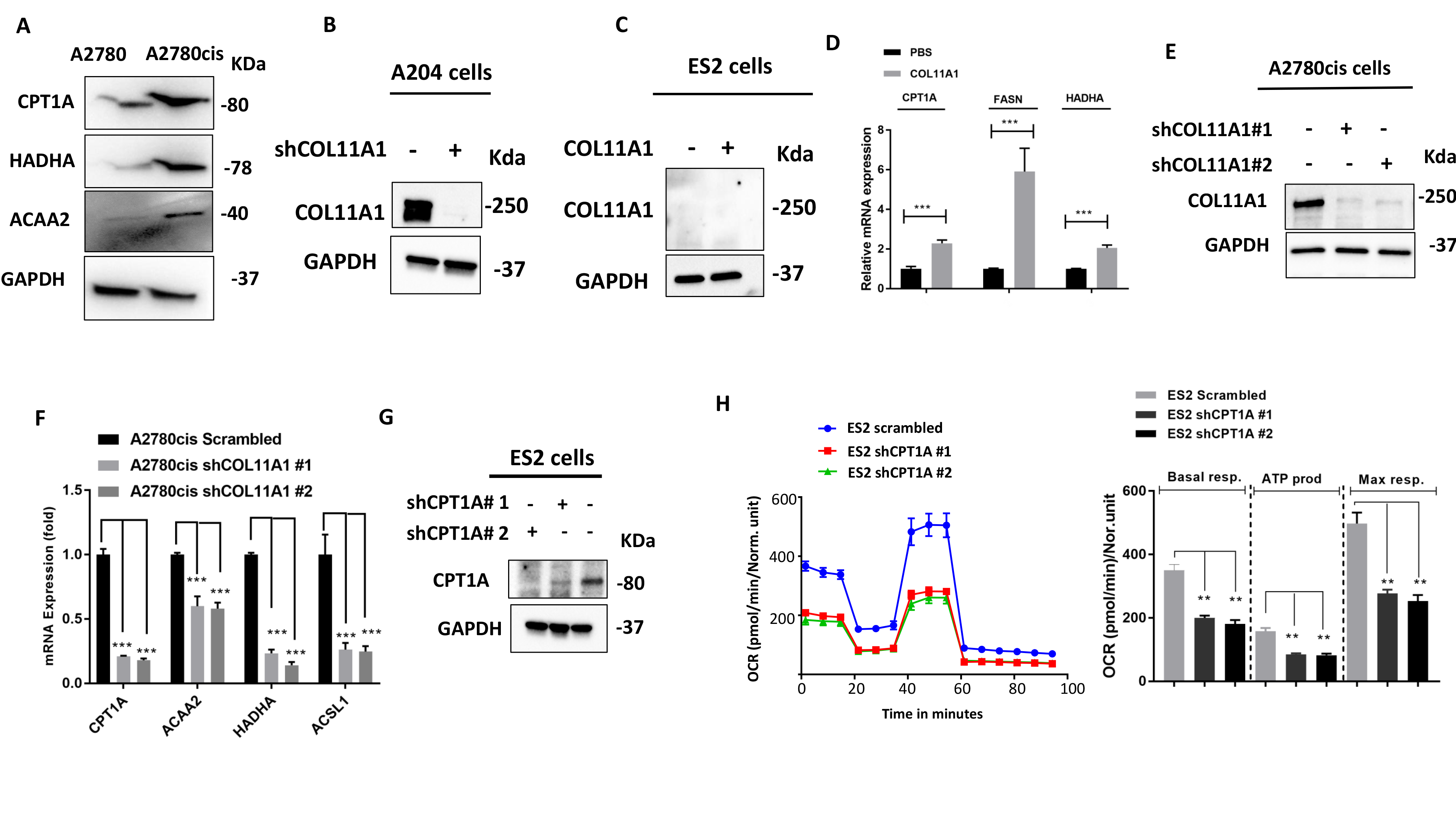

Supplement: Supplementary file 2 — Supplemental figure 1 [file 41419_2020_2442_MOESM2_ESM.tif]

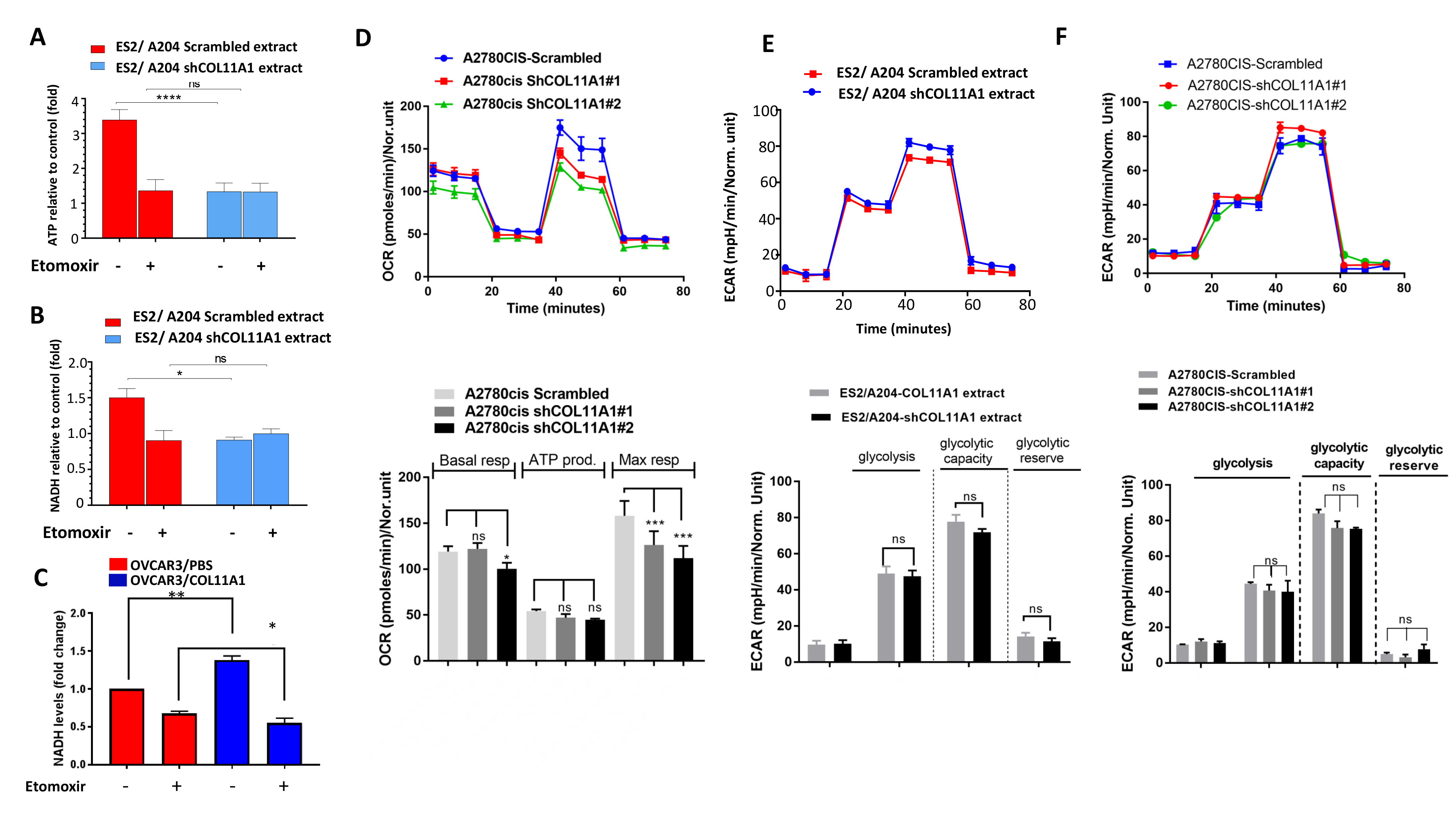

Supplement: Supplementary file 3 — Supplemental figure 2 [file 41419_2020_2442_MOESM3_ESM.tif]

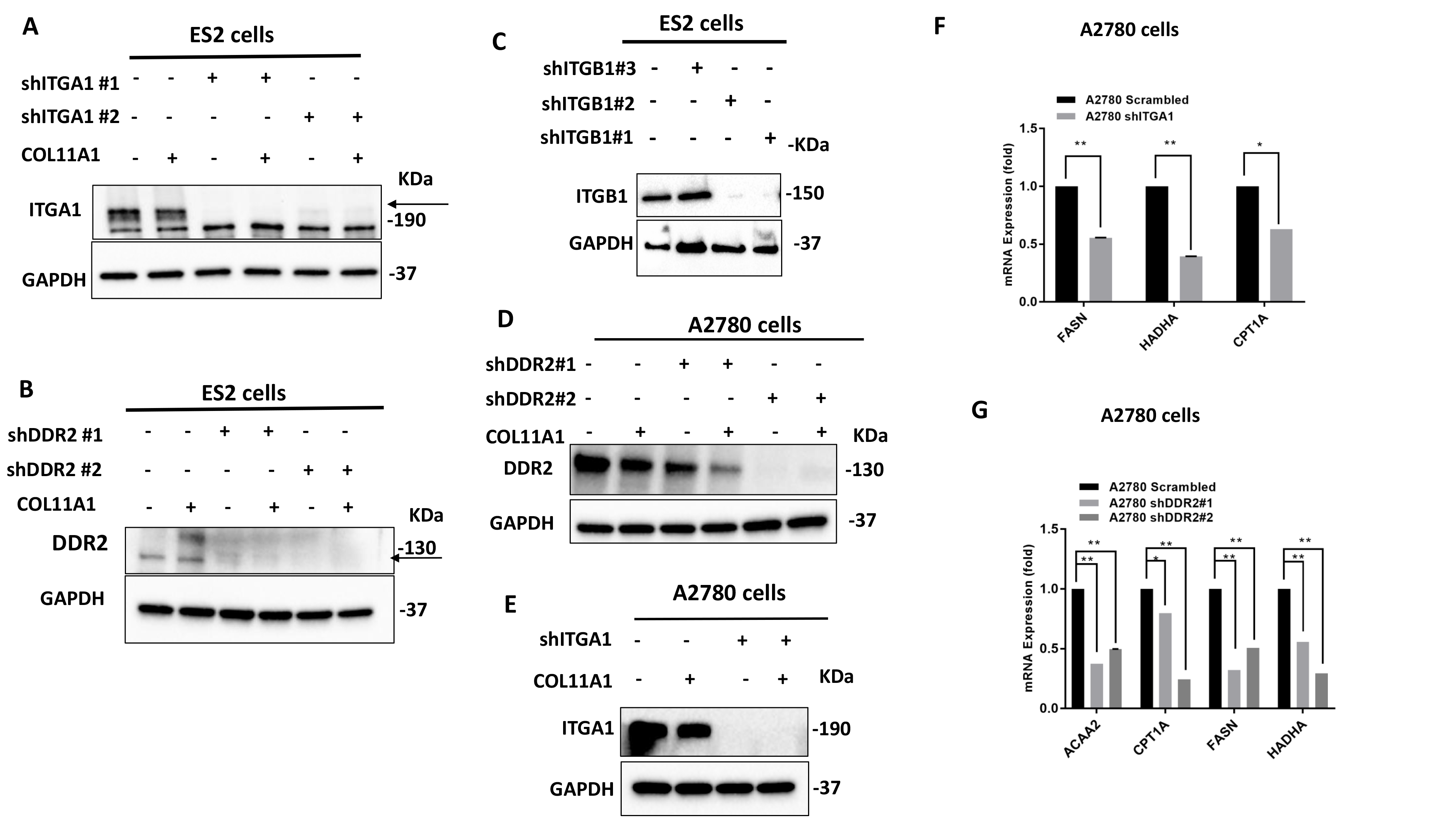

Supplement: Supplementary file 4 — Supplemental figure 3 [file 41419_2020_2442_MOESM4_ESM.tif]

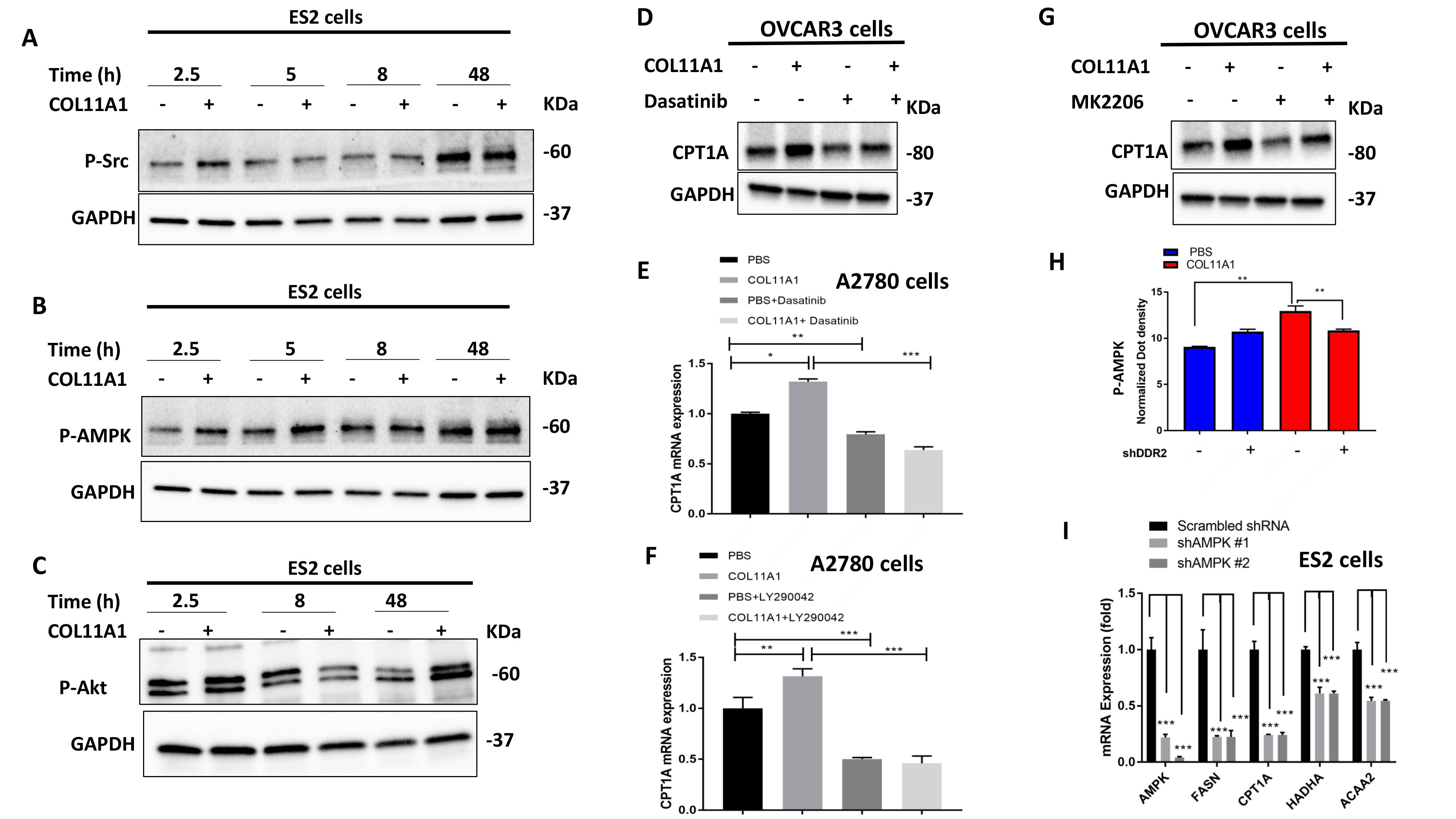

Supplement: Supplementary file 5 — Supplemental figure 4 [file 41419_2020_2442_MOESM5_ESM.tif]

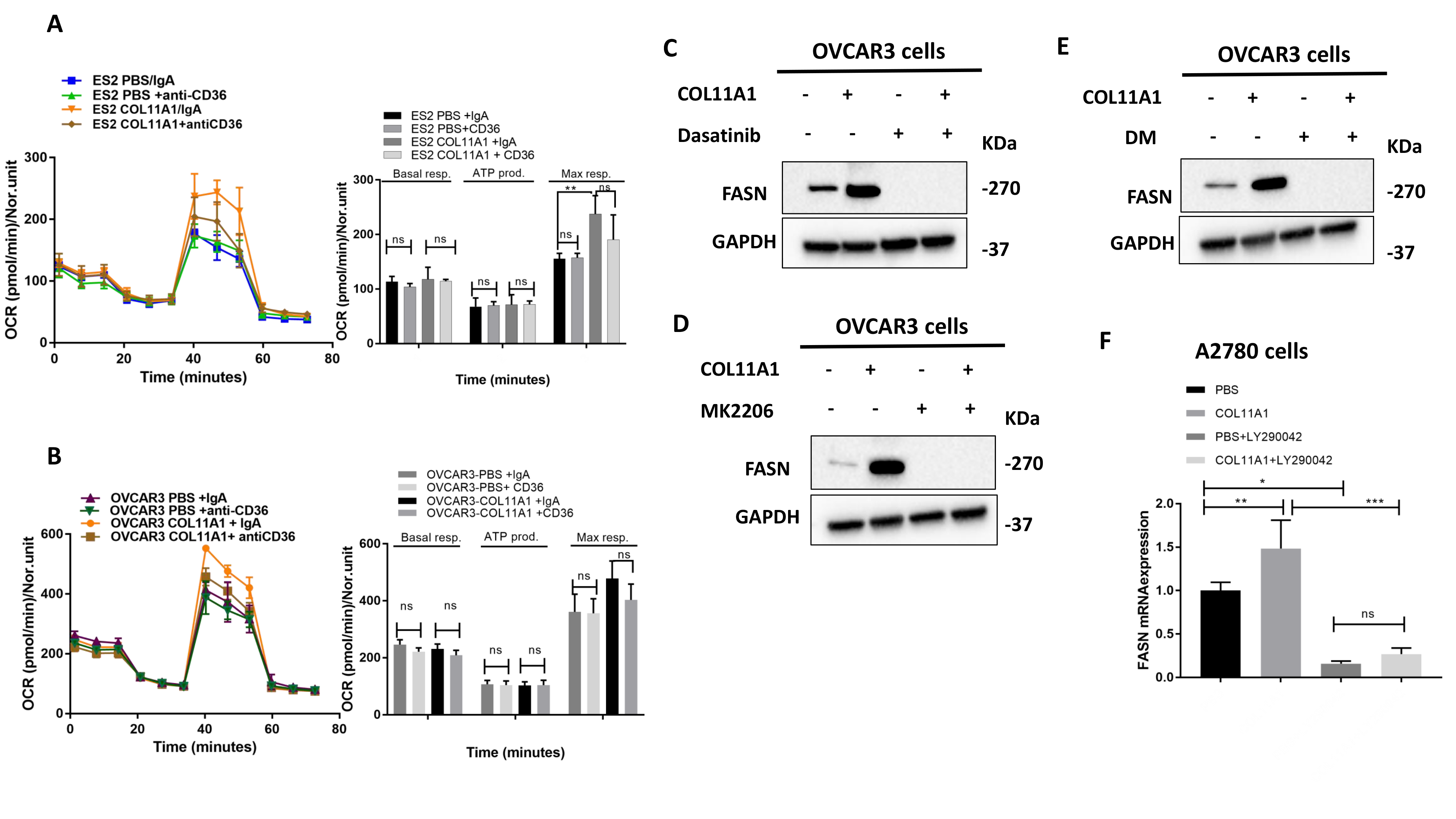

Supplement: Supplementary file 6 — Supplemental figure 5 [file 41419_2020_2442_MOESM6_ESM.tif]

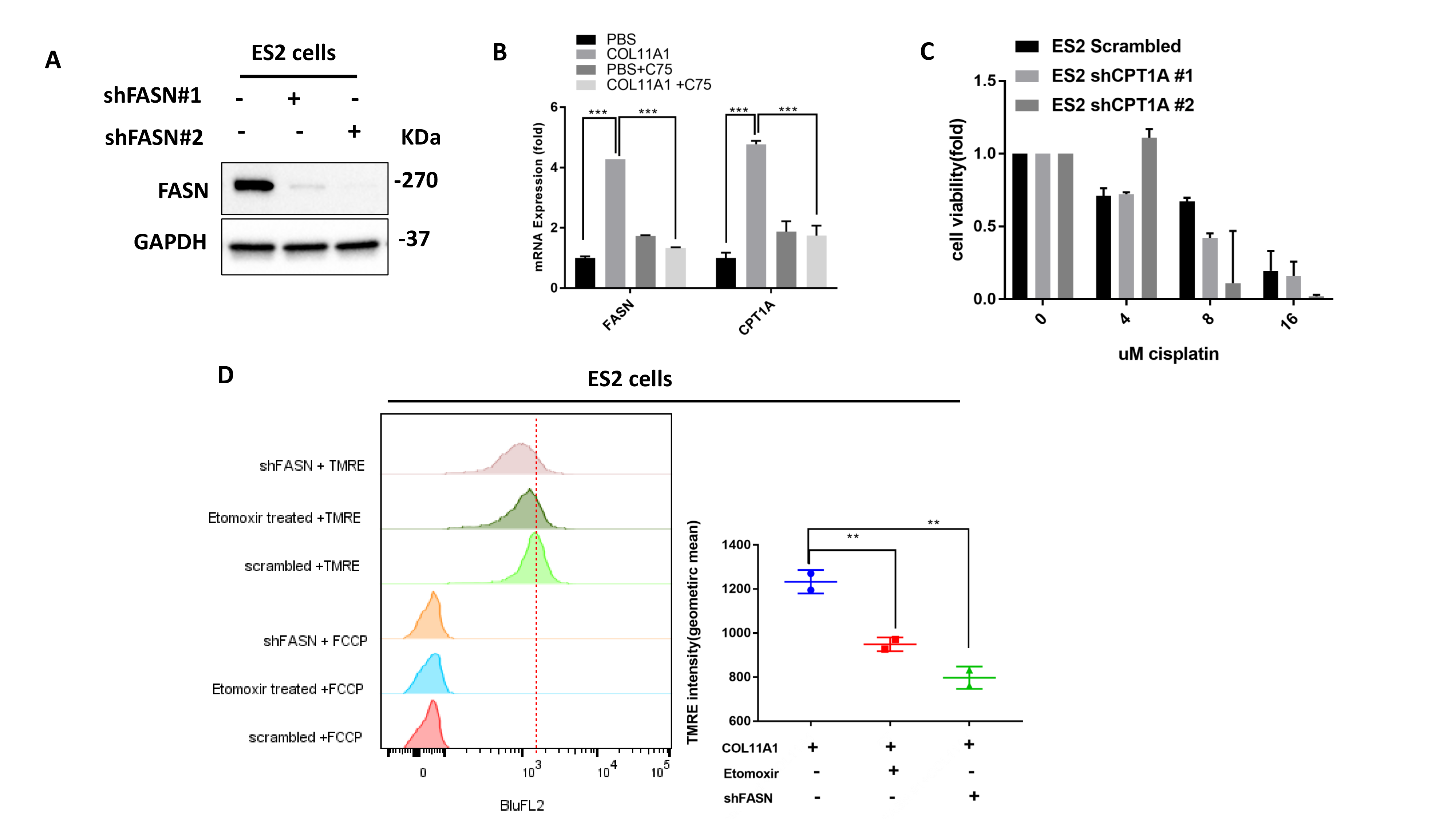

Supplement: Supplementary file 7 — Supplemental figure 6 [file 41419_2020_2442_MOESM7_ESM.tif]

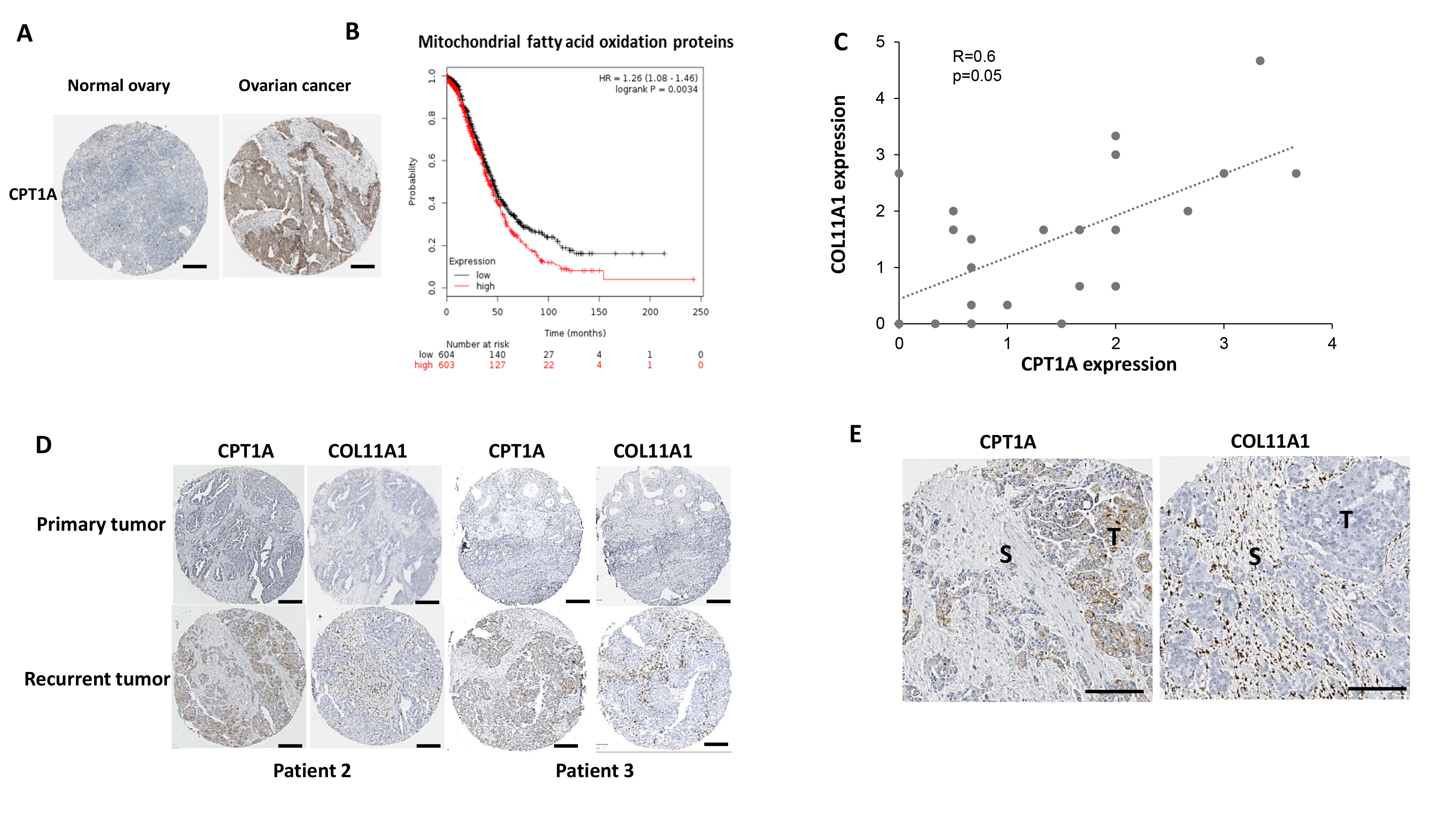

Supplement: Supplementary file 8 — Supplemental figure 7 [file 41419_2020_2442_MOESM8_ESM.tif]
